# Pretreatment monocyte counts and neutrophil counts predict the risk for febrile neutropenia in patients undergoing TPF chemotherapy for head and neck squamous cell carcinoma

**DOI:** 10.18632/oncotarget.24863

**Published:** 2018-04-10

**Authors:** Marie Shimanuki, Yorihisa Imanishi, Yoichiro Sato, Nana Nakahara, Daisuke Totsuka, Emiri Sato, Sena Iguchi, Yasuo Sato, Keiko Soma, Yasutomo Araki, Seiji Shigetomi, Satoko Yoshida, Kosuke Uno, Yusuke Ogawa, Takehiro Tominaga, Yuichi Ikari, Junko Nagayama, Ayako Endo, Koshiro Miura, Takuya Tomioka, Hiroyuki Ozawa, Kaoru Ogawa

**Affiliations:** ^1^ Department of Otorhinolaryngology–Head and Neck Surgery, Kawasaki Municipal Kawasaki Hospital, Kawasaki, Kanagawa, Japan; ^2^ Department of Otorhinolaryngology, Kyosai Tachikawa Hospital, Tachikawa, Tokyo, Japan; ^3^ Department of Otorhinolaryngology, Matsumoto Dental University, Matsumoto, Nagano, Japan; ^4^ Department of Otorhinolaryngology, Yokohama Municipal Citizen’s Hospital, Yokohama, Kanagawa, Japan; ^5^ Department of Otorhinolaryngology–Head and Neck Surgery, Keio University School of Medicine, Shinjuku, Tokyo, Japan; ^6^ Department of Otolaryngology–Head and Neck Surgery, National Defense Medical College, Tokorozawa, Saitama, Japan; ^7^ Department of Otorhinolaryngology, International University of Health and Welfare Atami Hospital, Atami, Shizuoka, Japan; ^8^ Department of Otorhinolaryngology, Saitama Red Cross Hospital, Saitama, Saitama, Japan; ^9^ Department of Otorhinolaryngology, Kamio Memorial Hospital, Chiyoda, Tokyo, Japan; ^10^ Department of Otorhinolaryngology, Ashikaga Red Cross Hospital, Ashikaga, Tochigi, Japan

**Keywords:** febrile neutropenia, TPF, monocyte count, neutrophil count, head and neck squamous cell carcinoma

## Abstract

**Background:**

Febrile neutropenia (FN) is the most serious hematologic toxicity of systemic chemotherapy. However, accurate prediction of FN development has been difficult because the risk varies largely depending on the chemotherapy regimen and various individual factors.

**Methods:**

We retrospectively analyzed diverse clinical factors including pretreatment hematological parameters to clarify the reliable predictors of FN development during chemotherapy with a docetaxel, cisplatin, and fluorouracil (TPF) regimen in patients with head and neck squamous cell carcinoma.

**Results:**

Among the 50 patients, grade ≥3 neutropenia, grade 4 neutropenia, and FN developed in 36 (72%), 21 (42%), and 12 (24%) patients, respectively. Multivariate logistic regression revealed that a pretreatment absolute monocyte count (AMC) <370/mm^3^ is an independent predictor of TPF chemotherapy-induced FN (odds ratio=6.000, p=0.017). The predictive performance of the model combining AMC and absolute neutrophil count (ANC), in which the high-risk group was defined as having an AMC <370/mm^3^ and/or ANC <3500/mm^3^, was superior (area under the curve [AUC]=0.745) to that of the model with a cutoff for AMC alone (AUC=0.679).

**Conclusions:**

On the basis of our results, we recommend primary prophylactic use of granulocyte colony-stimulating factor and/or antibiotics selectively for patients predicted to be at high risk for TPF chemotherapy-induced FN.

## INTRODUCTION

Neutropenia is one of the most common adverse effects of systemic anti-cancer chemotherapy and represents a major dose-limiting toxicity [1, 2]. In particular, febrile neutropenia (FN), a condition in which fever develops in the presence of neutropenia, is the most serious hematologic toxicity because it may predispose patients to life-threatening infections such as severe sepsis and septic shock [3, 4]. In unselected cohorts, the risk for FN-related mortality was estimated to be as high as 5–11% [5]. The management of FN requires intensive antibacterial therapy with broad-spectrum antibiotics and prolonged hospitalization, leading to treatment delays and dose reductions of chemotherapy that potentially compromise treatment outcomes [1–4]. If the patients who are at a high risk for FN can be distinguished appropriately before chemotherapy, efficient prevention of FN-induced serious infections can be feasible by precluding FN development via the administration of highly selective, prophylactic granulocyte colony-stimulating factor (G-CSF) to those at high risk for FN, as well as by avoiding unnecessary prescription of costly G-CSF and antibiotics to those at low risk for FN [4, 6, 7]. However, it has been difficult to accurately predict the development of FN, because the extent and timing of neutropenia, as well as susceptibility to infection, vary widely among individuals, which depends on not only chemotherapy regimens but also various patient factors.

With regards to induction chemotherapy for advanced head and neck squamous cell carcinoma (HNSCC), a docetaxel, cisplatin, and fluorouracil-based regimen (TPF) has been shown to be superior to others in randomized phase III trials wherein a larynx preservation strategy was attempted [8–12]. According to these clinical trials involving patients with HNSCC, the incidence of FN with regimens containing taxane and platinum ranges from 4.8% to 19% [8, 9, 13–16]. Nonetheless, in community settings, previous studies have shown that the incidence of FN in patients treated with taxane and platinum-based regimens was as high as 34–55%, and severe infections developed in 46–48% of the FN episodes [17, 18]. These results suggest that even within the same regimens, the risk for FN and its resulting complications differ largely according to the patients’ backgrounds and should be evaluated individually.

Previous studies on various malignancies have revealed that chemotherapy-induced FN involves diverse risk factors as follows: older age; poorer performance status; lower body weight; lower pretreatment blood cell counts that include white blood cells (WBCs), neutrophils, lymphocytes, and monocytes; presence of comorbidities involving major organs; advanced stage cancer; history of prior chemotherapy; higher dose intensity and number of cycles of chemotherapy [19–26]. However, differences in such significant predictors of FN among studies have largely depended on the cancer type and regimens applied. Concerning taxane and platinum-based regimens for patients with HNSCC, although only a few studies have been conducted, tube feeding, diabetes mellitus, and a high liver ultrasonography fibrotic score were identified as independent predictors of FN [17, 27]. Although pretreatment hematological parameters (i.e., WBC, neutrophil, lymphocyte, and monocyte counts) may directly reflect hematopoietic function and/or leukocyte counts in reservoirs within the body, no study has investigated the value of such hematological cell counts in the prediction of FN development exclusively in patients with HNSCC receiving TPF regimen.

We conducted this study to clarify the reliable predictors of FN development in patients with HNSCC who received TPF chemotherapy in a community setting by examining various clinical factors including pretreatment hematological cell counts.

## RESULTS

### Patient characteristics

The demographic and clinical characteristics of the 50 patients included in this study are summarized in Table 1. The median age was 65 years (range, 44–79 years), and the man-to-woman ratio was 22:3. The most common primary tumor site was the hypopharynx (n=25, 50%), followed by the oropharynx (n=9, 18%), larynx (n=6, 12%), and oro-hypopharynx (n=4, 8%). Most patients (n=45, 90%) had stage IV disease. The most common comorbidities were hypertension (n=19, 38%) and diabetes mellitus (n=5, 10%). Transnasal tube nutrition was initiated ahead of chemotherapy in 7 patients (14%) owing to difficulty in oral intake. The reason for TPF chemotherapy was induction in the majority of the patients (n=45, 90%), whereas that in the others (n=5, 10%) was first-line treatment for recurrent or metastatic disease.

**Table 1:** Patient characteristics (N=50)

Variables (continuous)	Median	(Range)
Age (yrs)	65	(44–79)
Height (cm)	165	(150.3–183.5)
Weight (kg)	58.8	(38.6–89.9)
BSA (m^2^)	1.64	(1.34–2.13)
BMI (kg/m^2^)	21.1	(14.9–29.4)

Variables (categorical)	No. of patients	%
Sex		
Men	44	88
Women	6	12
Primary tumor site		
Tongue	3	6
Oropharynx	9	18
Hypopharynx	25	50
Oro-hypopharynx	4	8
Larynx	6	12
Paranasal sinus	1	2
Unknown	2	4
T stage		
T0	6	12
T1	2	4
T2	4	8
T3	15	30
T4	23	46
N stage		
N0	8	16
N1	4	8
N2a	5	10
N2b	10	20
N2c	18	36
N3	5	10
M stage		
M0	45	90
M1	5	10
Stage		
I	0	0
II	2	4
III	3	6
IV	45	90
Smoking	45	90
Hypertension	19	38
Diabetes mellitus	5	10
Tube nutrition	7	14
Reason for chemotherapy		
Induction	45	90
First-line for R/M	5	10
Number of cycles		
1	18	36
2	26	52
3	3	6
4	3	6

BSA, body surface area; BMI, body mass index; R/M, recurrence or metastasis.

### Pretreatment laboratory data

The pretreatment hematological and biochemical data obtained prior to the first cycle of TPF chemotherapy are summarized in Table 2. For some variables, the total number of patients was less than 50 because some parameters had not been examined just before the first cycle in a few patients. The median values were used as the cutoff value for dichotomization of each variable in the following univariate analysis.

**Table 2:** Pretreatment hematological and biochemical laboratory data

Variables	Unit	N^a^	Median	(Range)
WBC	(/mm^3^)	50	6465	(3200–13600)
ANC	(/mm^3^)	47	4677	(2041–10350)
ALC	(/mm^3^)	47	1458	(470–2998)
AMC	(/mm^3^)	47	532	(210–1655)
Hemoglobin	(g/dL)	50	13.15	(8.9–16.2)
Platelet	(10^4^/mm^3^)	50	24.75	(12.6–56.2)
NLR		47	2.93	(0.88–10.68)
PLR		47	0.018	(0.006–0.054)
LMR		47	2.93	(0.71–10.30)
Total bilirubin	(mg/dL)	47	0.6	(0.2–2.2)
AST	(IU/L)	50	18.5	(5–115)
ALT	(IU/L)	50	14	(8–205)
LDH	(IU/L)	48	163	(109–403)
BUN	(mg/dL)	50	12	(3–24)
Creatinine	(mg/dL)	50	0.7	(0.4–1.2)
CCr^b^	(mL/min)	50	84.6	(50.1–168.7)
Albumin	(g/dL)	45	4.0	(2.8–4.6)
CRP	(mg/dL)	48	0.395	(0.01–7.02)

WBC, white blood cell; ANC, absolute neutrophil count; ALC, absolute lymphocyte count; AMC, absolute monocyte count; NLR, neutrophil-to-lymphocyte ratio; PLR, platelet-to-lymphocyte ratio; LMR, lymphocyte-to-monocyte ratio; AST, aspartate transaminase; ALT, alanine aminotransferase; LDH, lactate dehydrogenase ; BUN, blood urea nitrogen; CCr, creatinine clearance; CRP, C-reactive protein.

^a^Numbers of the patients were less than 50 in some variables.

^b^Calculated using Cockcroft-Gault equation.

### Incidence of FN and other adverse effects

The incidence of grade 3 or higher adverse effects in the first cycle of TPF chemotherapy are summarized in Table 3. FN developed in 12 of 50 patients (24%), while grade 3 or higher neutropenia was observed in 36 patients (72%); grade 4 neutropenia occurred in 21 patients (42%). Regarding non-hematological adverse effects, hyponatremia (n=22, 44%) and diarrhea (n=13, 26%) were most commonly observed. G-CSF was administered to 20 patients (40%) who developed FN or grade 4 neutropenia, of whom antibiotics were administered to 14 (28%) and treatment delay more than a week was necessitated due to severe infections in 3 patients (6%).

**Table 3:** Adverse effects in the first cycle of TPF chemotherapy (grade 3 or higher)

Adverse effects	Criteria^a^	No. of patients	%
Febrile neutropenia	(See footnote^b^)	12	24
Neutropenia	ANC <1000/mm^3^	36	72
Grade 4 neutropenia	ANC <500/mm^3^	21	42
Anemia	Hb <8.0 g/dL	3	6
Thrombocytopenia	Platelet <5.0 – 2.5 ×10^4^/mm^3^	1	2
Diarrhea	Increase of ≥7 stools per day over baseline	13	26
Hyponatremia	Na (sodium) <130 – 120 mmol/L	22	44
Hypokalemia	K (potassium) <3.0 – 2.5 mmol/L	4	8
Hyperkalemia	K (potassium) >6.0 – 7.0 mmol/L	1	2
ALT increased	>5.0 – 20.0 × ULN	1	2
Creatinine increased	>3.0 × baseline or >3.0 – 6.0 × ULN	2	4

ALT, alanine aminotransferase; ANC, absolute neutrophil count; Hb, Hemoglobin; ULN, upper limits of normal.

^a^According to the Common Terminology Criteria for Adverse Effects version 4.0, except for febrile neutropenia.

^b^A combination of an occurrence of a fever with axillary temperature ≥37.5°C and an ANC of <500/mm^3^ or that of <1000/mm^3^ with a predicted decline to <500/mm^3^ during the next 48 hours.

### Univariate and multivariate analyses of predictors of FN development

In the univariate analysis, we found that the pretreatment WBC count (P=0.044), absolute neutrophil count (ANC, P=0.020), and absolute monocyte count (AMC, P=0.049) were significantly correlated with FN development in the first cycle of TPF chemotherapy (Table 4). On the other hand, the other variables including age, body mass index, tumor-related stage, comorbidities, tube nutrition, absolute lymphocyte count, anemia, and hypoalbuminemia, showed no significant correlation with FN development.

**Table 4:** Univariate analysis of predictive factors of FN development

Variables		No. of patients^a^		P value
		FN+	FN-	
Age (yrs)	≥65	7	20	1.000
	<65	5	18	
Sex	Men	12	32	0.314
	Women	0	6	
Height (cm)	≥165	7	20	1.000
	<165	5	18	
Weight (kg)	≥58.8	7	19	0.745
	<58.8	5	19	
BSA (m^2^)	≥1.64	6	19	1.000
	<1.64	6	19	
BMI (kg/m^2^)	≥21.1	6	20	1.000
	<21.1	6	18	
Primary tumor site	Hypopharynx	7	22	1.000
	Others	5	16	
T stage	0–3	7	20	1.000
	4	5	18	
N stage	0	4	5	0.191
	≥1	8	33	
M stage	0	10	35	0.582
	1	2	3	
Stage	I–III	0	5	0.319
	IV	12	33	
Brinkman index	≥820	4	23	0.183
	<820	8	15	
Hypertension	Yes	3	16	0.332
	No	9	22	
Diabetes mellitus	Yes	0	5	0.319
	No	12	33	
Tube nutrition	Yes	0	7	0.174
	No	12	31	
Reason for chemotherapy	Induction	11	34	1.000
	For R/M	1	4	
WBC (/mm^3^)	≥6465	3	24	0.044
	<6465	9	14	
ANC (/mm^3^)	≥4677	3	23	0.020
	<4677	9	12	
ALC (/mm^3^)	≥1458	6	20	0.744
	<1458	6	15	
AMC (/mm^3^)	≥532	3	21	0.049
	<532	9	14	
Hemoglobin (g/dL)	≥13.15	9	17	0.100
	<13.15	3	21	
Platelet (10^4^/mm^3^)	≥24.75	5	22	0.508
	<24.75	7	16	
NLR	≥2.93	5	20	0.505
	<2.93	7	15	
PLR	≥0.018	5	20	0.505
	<0.018	7	15	
LMR	≥2.93	7	17	0.740
	<2.93	5	18	
Total bilirubin (mg/dL)	≥0.6	7	18	0.747
	<0.6	5	17	
AST (IU/L)	≥18.5	4	21	0.321
	<18.5	8	17	
ALT (IU/L)	≥14	6	19	1.000
	<14	6	19	
LDH (IU/L)	≥163	6	20	0.751
	<163	6	16	
BUN (mg/dL)	≥12	5	23	0.324
	<12	7	15	
Creatinine (mg/dL)	≥0.7	7	20	1.000
	<0.7	5	18	
CCr (mL/min)	≥84.6	8	17	0.321
	<84.6	4	21	
Albumin (g/dL)	≥4.0	7	19	0.736
	<4.0	4	15	
CRP (mg/dL)	≥0.395	4	20	0.318
	<0.395	8	16	

FN, febrile neutropenia; BSA, body surface area; BMI, body mass index; R/M, recurrence or metastasis; WBC, white blood cell; ANC, absolute neutrophil count; ALC, absolute lymphocyte count; AMC, absolute monocyte count; NLR, neutrophil-to-lymphocyte ratio; PLR, platelet-to-lymphocyte ratio; LMR, lymphocyte-to-monocyte ratio; AST, aspartate transaminase; ALT, alanine aminotransferase; LDH, lactate dehydrogenase ; BUN, blood urea nitrogen; CCr, creatinine clearance; CRP, C-reactive protein.

^a^Numbers of the patients in total were less than 50 in some variables of hematological and biochemical laboratory data as shown in Table 2.

**Figure 1:**
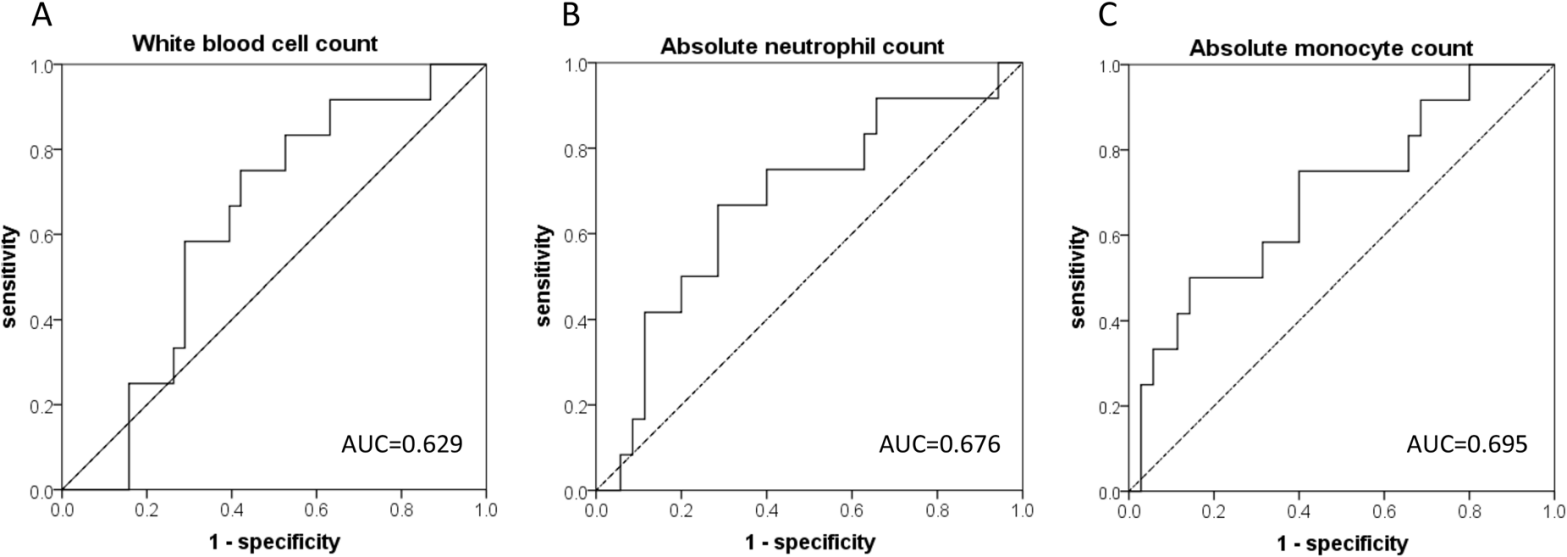
The receiver operator characteristic (ROC) curves for the prediction of febrile neutropenia (FN) development for **(A)** pretreatment white blood cell count (WBC), **(B)** absolute neutrophil count (ANC), and **(C)** absolute monocyte count (AMC). The areas under the curve (AUC) were 0.629 for WBC, 0.676 for ANC, and 0.695 for AMC.

Using receiver operating characteristic (ROC) curves, we determined the optimal cutoff values of the above-mentioned hematological variables as follows: 6500/mm^3^ for WBC, 3500/mm^3^ for ANC, and 370/mm^3^ for AMC; the area under the curve (AUC) for each variable is shown in Figure 1. The multivariate logistic regression model revealed that AMC <370/mm^3^ was the only independently significant predictor of TPF chemotherapy-induced FN (odds ratio=6.000 [95% confidence interval: 1.372–26.237] vs. AMC ≥370/mm^3^, P=0.017, Table 5 ).

**Table 5:** Logistic regression model of predictive factors of FN development

Step	Covariate	Dichotomization	Odds ratio	95% confidence interval	P value
1	WBC	(<6500/mm^3^ vs. ≥6500/mm^3^)	0.999	(0.068–14.788)	1.000
	ANC	(<3500/mm^3^ vs. ≥3500/mm^3^)	3.757	(0.303–46.643)	0.303
	AMC	(<370/mm^3^ vs. ≥370/mm^3^)	4.427	(0.885–22.150)	0.070
					
2	ANC	(<3500/mm^3^ vs. ≥3500/mm^3^)	3.755	(0.851–16.557)	0.081
	AMC	(<370/mm^3^ vs. ≥370/mm^3^)	4.426	(0.935–20.949)	0.061
					
3	AMC	(<370/mm^3^ vs. ≥370/mm^3^)	6.000	(1.372–26.237)	0.017

FN, febrile neutropenia; WBC, white blood cell; ANC, absolute neutrophil count; AMC, absolute monocyte count**.**

### Diagnostic performance of the prediction of FN development

**Figure 2:**
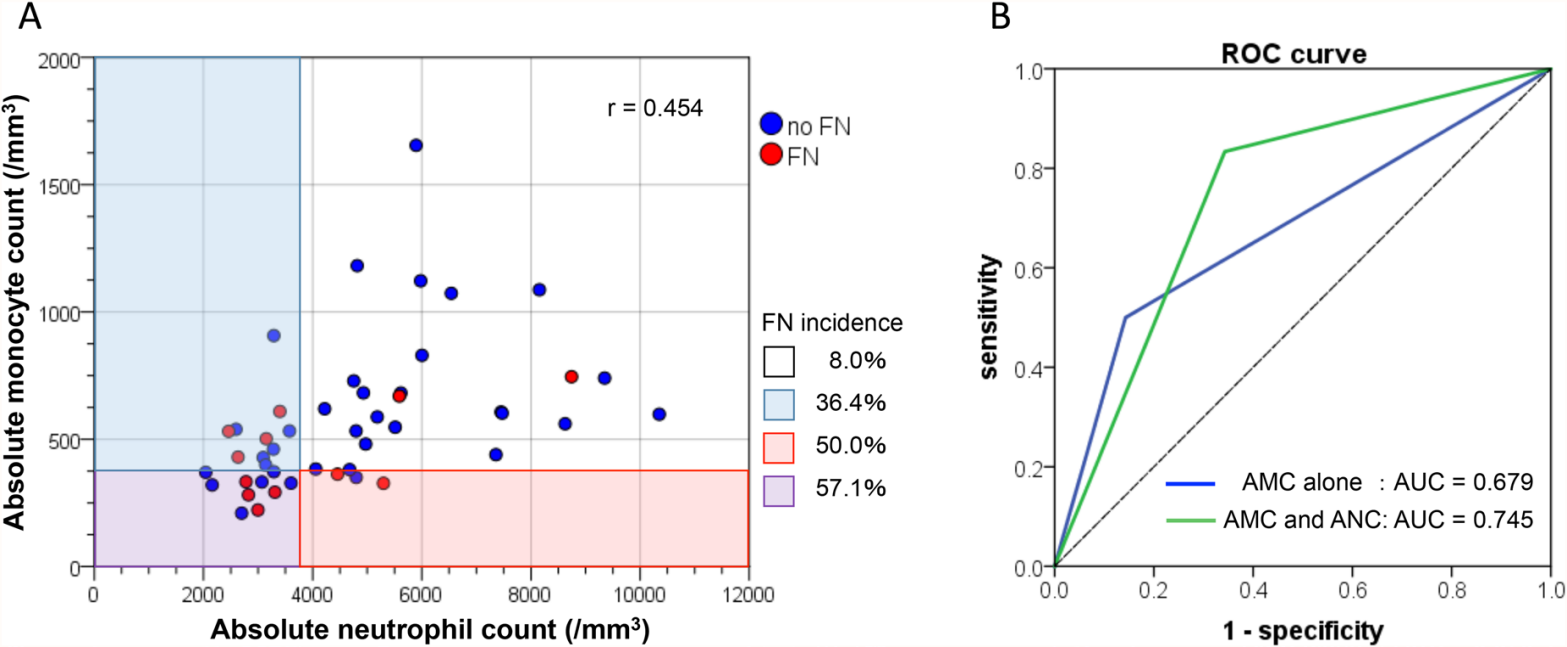
**(A)** The distribution diagram of absolute monocyte count (AMC) and absolute neutrophil count (ANC). Red circles indicate the patients who developed febrile neutropenia (FN) and blue circles indicate those without FN, which suggest a moderate linear association with a correlation coefficient of r=0.454 (P=0.001). **(B)** A comparison of receiver operator characteristic (ROC) curves between AMC alone (blue, AUC=0.679) and a combination of AMC and ANC (green, AUC=0.745) using the optimal cutoff value of those variables.

To assess the clinical usefulness of the aforementioned significant predictors, we compared the diagnostic performance for the prediction of FN development between AMC alone and a combination of AMC and ANC in the evaluable 47 patients, by dichotomizing them into two groups (high-risk group vs. low-risk group) using the cutoff value of those variables as shown in Table 6. When an AMC of 370/mm^3^ was used as the cutoff value, the FN incidences in the high- and low-risk groups were 54.5% and 16.7%, respectively (P=0.020). The sensitivity, specificity, positive likelihood ratio, negative likelihood ratio, and odds ratio in this model for prediction of FN development were 50.0%, 85.7%, 3.50, 0.58, and 6.00, respectively. On the other hand, when the low-risk group was defined as having an AMC ≥370/mm^3^ and ANC ≥3500/mm^3^ and the high-risk group was defined as the remainders (i.e., having an AMC <370/mm^3^ and/or ANC <3500/mm^3^), the FN incidences in the high- and low-risk groups were 45.5% and 8.0%, respectively (P=0.006). The sensitivity, specificity, positive likelihood ratio, negative likelihood ratio, and odds ratio in this model were 83.3%, 65.7%, 2.43, 0.25, and 9.58, respectively. The distribution diagram of AMC and ANC showed a moderate linear association between AMC and ANC, with a correlation coefficient of r=0.454 (P=0.001; Figure 2A). According to the ROC curves, the AUC of the latter model in which the cutoff was a combination of AMC and ANC was greater than that of the former model in which the cutoff involved AMC alone (0.745 vs. 0.679, Figure 2B).

**Table 6:** Comparison of predictive performance of FN development between AMC and AMC+ANC (N=47)

Variables	AMC					AMC+ANC				
Dichotomization	High risk: AMC <370/mm^3^ Low risk: AMC ≥370/mm^3^					High risk: AMC <370/mm^3^ and/or ANC <3500/mm^3^ Low risk: AMC ≥370/mm^3^ and ANC ≥3500/mm^3^				
	AMC	FN+	FN-	Total	FN incidence	AMC+ANC	FN+	FN-	Total	FN incidence
Two by two contingency tables	High risk	6	5	11	54.5%	High risk	10	12	22	45.5%
	Low risk	6	30	36	16.7%	Low risk	2	23	25	8.0%
	Total	12	35	47		Total	12	35	47	
Fisher’s exact test	P=0.020					P=0.006				
Diagnostic performance	Value		(95% CI)			Value		(95% CI)		
Sensitivity	50.0%		(28.5–68.5%)			83.3%		(59.3–95.1%)		
Specificity	85.7%		(78.3–92.1%)			65.7%		(57.5–69.8%)		
Positive predictive value	54.5%		(31.1–74.8%)			45.5%		(32.4–51.9%)		
Negative predictive value	83.3%		(76.2–89.5%)			92.0%		(80.5–97.7%)		
Positive likelihood ratio	3.50		(1.32–8.64)			2.43		(1.40–3.14)		
Negative likelihood ratio	0.58		(0.34–0.91)			0.25		(0.07–0.71)		
Odds ratio	6.00		(1.44–25.26)			9.58		(1.97–44.80)		
AUC	0.679		(0.488–0.869)			0.745		(0.588–0.903)		

FN, febrile neutropenia; AMC, absolute monocyte count; ANC, absolute neutrophil count; CI, confidence interval; AUC, area under the curve.

## DISCUSSION

In the present study, we found that neutropenia was the most frequent grade 3 or higher adverse effect (72%) to occur in the cohort of patients with HNSCC who underwent TPF chemotherapy. Notably, the incidence of FN was 24%, which is consistent with that reported in recent studies conducted in a community setting similar to ours [27, 28], but apparently higher than those observed in clinical trials (4.8–19%) [8, 9, 13–16]. This inconsistency is likely attributable to differences in the backgrounds of the patient cohorts, because those selected for clinical trials inevitably tend to be in better health since they are younger, and demonstrate a better performance status and no severe comorbidities or history of prior cancer treatments. In addition, even within a community setting, much higher incidences of FN (34–55%) have also been reported in previous studies wherein the majority of the patients treated with taxane and platinum-based chemotherapy already had received prior chemotherapy and radiotherapy [17, 18]. These observations suggest a limitation of a regimen-based prediction of chemotherapy-induced FN and the importance of the addition of an individual characteristics-based prediction strategy in real-world clinical practice.

The present study, in which we assessed the association between pretreatment hematological parameters and FN development exclusively in patients with HNSCC for the first time, demonstrated that pretreatment AMC is an independent risk factor predictive of FN development in HNSCC patients who underwent TPF chemotherapy. Moreover, although the correlation between pretreatment ANC and FN development was not shown to be independently significant, the prediction model comprising a combination of AMC and ANC in which the high-risk group was defined as having an AMC <370/mm^3^ and/or ANC <3500/mm^3^ demonstrated a superior diagnostic performance in the prediction of FN development when compared to that of the model comprising AMC alone. Even in terms of cancers other than HNSCC, only a limited number of previous studies have examined the correlation between pretreatment AMC and FN development using a multivariate analysis [20, 21, 26]. Among them, in accordance with our results, Moreau et al. reported that a baseline monocyte count <150/mm^3^ is one of the independent predictors of FN in patients with hematological malignancies to whom more than 100 regimens were administered [21]. However, two other studies showed no correlation between the pretreatment AMC and FN development in patients with various hematological and solid tumors [20, 26]. Another study reported that incorporating the pretreatment AMC into a FN prediction model that consists of pretreatment ANC and the absolute lymphocyte count could improve the predictive ability, even though the pretreatment AMC alone showed no significant association with FN, in patients with early-stage breast cancer [29]. These inconsistent results are thought to be attributable to variances not only in the patients’ background, including the type of cancer, but also in the regimens applied.

Regarding the physiologic mechanism of hematopoiesis, both neutrophils and monocytes differentiate from common myeloid progenitor cells, granulocyte–macrophage (GM) colony forming cells derived from multipotential hematopoietic stem cells, whereas lymphocytes have no direct progenitors in common with neutrophils [30]. This differentiation system can in part explain the close association between neutropenia and monocytopenia induced during chemotherapy. In addition, monocytes play a key role in promoting proliferation, differentiation, survival, and activation of both neutrophils and monocytes/macrophages themselves by producing GM-CSF, G-CSF, macrophage-CSF, and other cytokines, in both paracrine and autocrine manners [31–34]. These underlying mechanisms can account for why decreased pretreatment AMC indirectly reflects lower hematopoietic function of the bone marrow that inevitably worsens the extent of chemotherapy-induced neutropenia. Monocytes and macrophages play critical roles in the innate immune system principally through phagocytosis and the release of inflammatory cytokines, and also help initiate the acquired immune system response by presenting antigens to recruit other immune cells such as lymphocytes [35, 36]. Thus, decreased pretreatment AMC may also reflect suppressed immune function that can increase the susceptibility to infection. Taken together, lower pretreatment AMC is thought to be predictive of FN development presumably through a subclinical decline in both hematopoietic function and immune function.

During the course of chemotherapy-induced leukocytopenia, AMC is empirically known to precede ANC in terms of its onset, nadir, and recovery, as recently confirmed by Moriyama et al. who also reported a moderate correlation between AMC at the preceding nadir and ANC at the following nadir during platinum-based chemotherapy for lung cancer patients [37]. This phenomenon can be explained in part by the study that investigated the difference in the cell division history between these cells during their differentiation using a murine model, in which GM progenitors were demonstrated to generate postmitotic monocytes earlier than mature neutrophils [38]. Concerning the predictive ability of the post-treatment monocyte count measured during the early phases of chemotherapy, Kondo et al. first reported that an AMC <150/mm^3^ on days 6 to 8 is a possible predictor of grade 3 or 4 neutropenia during cisplatin-based chemotherapy for advanced lung cancer [39], while Oguz et al. revealed an AMC ≤150/mm^3^ on day 7 is an independent risk factor for FN in various chemotherapy regimens for childhood solid tumors [40]. In support of these results, by analyzing the post-treatment hematological data in the same cohort of the present study, we also found that an AMC ≤66/mm^3^ on days 3 to 5 is an independent risk factor of FN (data not shown). Nonetheless, considering the possible preparation required to prevent FN such as dose reduction of anti-cancer drugs and prophylactic use of antibiotics and/or G-CSF, the assessment of FN risk before chemotherapy using pretreatment information is thought to be more practical than that after chemotherapy using post-treatment information.

Neutrophils play a major role in the front-line host defense against invading micro-organisms by directly attacking pathogens via three mechanisms: phagocytosis, degranulation (release of soluble anti-microbials), and generation of neutrophil extracellular traps, in addition to recruiting and activating other immune cells by releasing various cytokines [41]. Therefore, decreased pretreatment ANC is also thought to reflect insufficiency in immune function as well as hematopoietic function, which could be predictive of FN development. However, among the previous studies that examined the predictive ability of pretreatment ANC, rather interestingly, only a few studies found pretreatment ANC to be a predictive factor of FN development [21, 42] or initial hospitalization due to FN [43] at least in the univariate analysis in line with our result, whereas other studies showed no association between them [26, 29, 44–48]. Neutrophils have been known to be activated and/or to increase in cell count in response to not only bacterial infection but also various other conditions including tissue injury after ischemia, administration of corticosteroids, hemodialysis, and smoking [41, 49–51], which may underestimate the potential suppression of hematopoietic and/or immune function. Hence, variances in the extent of heterogeneity of those comorbidity factors among the study cohorts could be a feasible explanation for the aforementioned inconsistent results. Namely, because those factors were relatively homogeneous in our cohort, in which no patients had co-morbidities that would induce noticeable neutrophilia except for a few patients while the vast majority had a smoking habit, pretreatment ANC in our study was thought to more evenly reflect hematopoietic and immune functions. Regardless of aforementioned variances among patient cohorts, we must be cautious when interpreting the ANC value because of its complex regulatory mechanisms as discussed above. Even if ANC remains within the normal range, we should be aware of the possible co-existence of both declined hematopoietic and/or immune function resulting in neutropenia and co-morbidities leading to reactive neutrophilia, since these can offset one another’s effect on ANC and/or WBC count.

According to three previous studies that focused exclusively on FN in HNSCC patients who received a docetaxel plus platinum-based chemotherapy, tube feeding, diabetes mellitus [17], and a high liver ultrasound fibrotic score [27] were demonstrated as independent factors predictive of FN, while tube feeding and a higher modified Charlson co-morbidity index were shown as independent predictors of severe infections [18]. In general, patients with locally advanced HNSCC tend to have difficulty with oral ingestion because of the close association between the anatomical location and swallowing function, which further tends to result in poor nutritional status (e.g., hypoalbuminemia) and necessity of tube feeding. However, such factors were not found to be predictive of FN in the present study, inconsistent with the previous studies conducted within a community setting [17, 18]. Tube feeding is known to deteriorate the risk for diarrhea and aspiration pneumonia, but otherwise can stably provide adequate nutrition. Therefore, the possible disparity in the resultant imbalance between such undesired risks and beneficial effects among the studies could in part explain the above-mentioned inconsistent results. Moreover, although diabetes mellitus and other comorbidities were not found to be predictors of FN in our study either, such inconsistency could be attributed to differences in the extent of severity of those diseases and how they were appropriately controlled before chemotherapy.

In our cohort, G-CSF was administered in principle for therapeutic purposes (in patients with severe neutropenia or FN), because its prophylactic use had not been approved until 2013 per the Japanese medical insurance system. In our experience, therapeutic administration of G-CSF still fortunately has been able to prevent a majority of the patients with FN from developing serious infections such as severe sepsis and septic shock, although this type of G-CSF use has not been recommended by international guidelines [15, 52]. Since the Japanese practice guidelines were also revised in 2013 to allow for the use of primary prophylactic G-CSF in chemotherapy regimens wherein the risk of FN incidence is over 20% [53], currently, primary prophylaxis may be permissible in Japan as long as the patients undergo TPF chemotherapy in a community setting. Nonetheless, in terms of the cost-effectiveness, unconditional prophylactic use of expensive G-CSF in all these patients would not be ideal despite the high risk (20–30%) of FN incidence. Hence, it is crucial to identify patients at higher risk for FN prior to chemotherapy and to prevent them from developing FN more efficiently via selective prophylactic administration of G-CSF. On the basis of the present results, we recommend primary prophylaxis against FN via the administration of G-CSF selectively to patients predicted to be at high risk for TPF chemotherapy-induced FN according to a dependable prediction model.

There are several limitations in our study, most of which are due to its retrospective nature. First, even in a community setting, patients with a poor performance status or serious medical illnesses were not scheduled to receive the TPF regimen although the criteria were not as strict as those of the clinical trials, which may lead to bias in terms of patient selection. Even within the patients who underwent TPF chemotherapy, the dose was reduced owing to hepatic or renal disorders. Therefore, our results may have underestimated the risk for TPF chemotherapy-induced FN. Second, because of the relatively small sample size in our cohort, the statistical power might not be enough to identify the significance of other possible predictive factors. However, considering the well-known wide variance in predictors across different regimens, it is valuable to elucidate the predictors of FN development exclusively in patients receiving the TPF regimen. Third, since we failed to examine some hematological parameters just before the first cycle in a few patients, missing values remained in several variables, which might have resulted in a biased data collection. Fourth, because we did not validate our prediction model using an external dataset, a prospective trial or a validation study will be indispensable to corroborate the reliability of its predictive performance.

In conclusion, the present study demonstrated that a pretreatment AMC <370/mm^3^ is an independent predictor of TPF chemotherapy-induced FN in patients with HNSCC. In addition, the prediction model comprising a combination of pretreatment AMC and ANC, in which the high-risk group was defined as having an AMC <370/mm^3^ and/or ANC <3500/mm^3^, yielded a superior predictive performance. Therefore, primary prophylaxis for FN via administration of G-CSF and/or antibiotics should be selectively considered for patients at high risk for FN according to the aforementioned prediction model.

## PATIENTS AND METHODS

### Patients and data collection

We retrospectively reviewed the medical records of patients with HNSCC who received TPF chemotherapy from January 2010 through December 2016 at the Department of Otorhinolaryngology–Head and Neck Surgery, Kawasaki Municipal Kawasaki Hospital. The Institutional Review Board and Research Ethics Committee approved the study protocol (reference numbers: 2017-10). The requirement for informed consent was waived owing to the retrospective nature of the analysis.

In total, 52 patients underwent TPF chemotherapy at least once or more during the aforementioned period. Two patients were excluded from the analysis because of prophylactic use of G-CSF in the first cycle of TPF chemotherapy in consideration of the apparently high probability of FN. The remaining 50 patients were enrolled in the study. We extracted the information that could be associated with development of FN from the medical charts. Patient characteristics included age, sex, body height, body weight, body mass index, and smoking status (Brinkman index). Disease characteristics included primary tumor site, TNM stage according to the UICC TNM classification and staging system (7th edition, 2010), and co-existing morbidities. Treatment factors included reason for chemotherapy and necessity of tube nutrition. Pretreatment hematological and biochemical data were obtained from laboratory blood examination tests performed within 2 weeks before the first cycle of the chemotherapy. The neutrophil-to-lymphocyte ratio, platelet-to-lymphocyte ratio, and lymphocyte-to-monocyte ratio were calculated by dividing absolute values of the corresponding blood cell counts.

### TPF chemotherapy and adverse effect evaluation

All patients were administered a 1-hour intravenous infusion of docetaxel (60 mg/m^2^) on day 1, a 3-hour intravenous infusion of cisplatin (60 mg/m^2^) on day 1, and a continuous 24-hour infusion of fluorouracil (700 mg/m^2^) on days 1-4, except for 4 patients who required dose reduction because of hepatic or renal disorders. All patients were hospitalized for approximately 3 weeks to undergo TPF chemotherapy together with supportive care for the related adverse effects. Laboratory blood tests were performed twice or three times per week to identify hematopenia including neutropenia, electrolyte imbalance, and other adverse effects.

Chemotherapy-related adverse effects were evaluated according to the Common Terminology Criteria for Adverse Effects version 4.0, except for FN. For the purpose of this study, we defined FN as a combination of an occurrence of a fever with an axillary temperature ≥37.5°C and a neutrophil count of <500/mm^3^ or that of <1000/mm^3^ with a predicted decline to <500/mm^3^ during the next 48 hours, as suggested by the Japanese Society of Medical Oncology, because body temperature is measured under the axilla in Japan and the axillary temperature is approximately 0.5°C below the oral temperature. We also documented the use of G-CSF and antibiotics, which were not for primary prophylactic purposes but for therapeutic purposes only.

### Statistical analysis

Patient characteristics, disease characteristics, treatment factors, and pretreatment hematological and biochemical data were examined for their correlation with the development of chemotherapy-induced FN in the first cycle using the two-tailed Fisher’s exact test. The continuous variables were dichotomized with the cutoff values using the median values. The independent significance of the variables detected to be significant in the univariate analysis was further assessed via a multivariate logistic regression analysis with the backward stepwise selection method, in which the optimal cutoff values were determined using the Youden index of the ROC curve. The diagnostic performance of the significant variables in the prediction of FN development was evaluated using two by two contingency tables and the AUC determined on the basis of the ROC curve. A linear association between paired continuous variables was evaluated using Pearson's correlation coefficient. P-values <0.05 were considered statistically significant. All analyses were carried out using SPSS version 24.0 (SPSS, Chicago, IL).

## Abbreviations

FN: febrile neutropenia
TPF: docetaxel, cisplatin, and fluorouracil
AMC: absolute monocyte count
ANC: absolute neutrophil count
G-CSF: granulocyte colony-stimulating factor
HNSCC: head and neck squamous cell carcinoma
WBC: white blood cells
ROC: receiver operating characteristic
AUC: area under the curve
GM: granulocyte–macrophage
